# How do individuals’ health behaviours respond to an increase in the supply of health care? Evidence from a natural experiment

**DOI:** 10.1016/j.socscimed.2016.05.005

**Published:** 2016-06

**Authors:** Eleonora Fichera, Ewan Gray, Matt Sutton

**Affiliations:** Manchester Centre for Health Economics, University of Manchester, United Kingdom

**Keywords:** England, Health behaviours, Healthcare supply, Quality and outcomes framework, Financial incentives, Spillovers, Regression discontinuity

## Abstract

The efficacy of the management of long-term conditions depends in part on whether healthcare and health behaviours are complements or substitutes in the health production function. On the one hand, individuals might believe that improved health care can raise the marginal productivity of their own health behaviour and decide to complement health care with additional effort in healthier behaviours. On the other hand, health care can lower the cost of unhealthy behaviours by compensating for their negative effects. Individuals may therefore reduce their effort in healthier lifestyles. Identifying which of these effects prevails is complicated by the endogenous nature of treatment decisions and individuals’ behavioural responses. We explore whether the introduction in 2004 of the Quality and Outcomes Framework (QOF), a financial incentive for family doctors to improve the quality of healthcare, affected the population’s weight, smoking and drinking behaviours by applying a sharp regression discontinuity design to a sample of 32,102 individuals in the Health Survey for England (1997–2009). We find that individuals with the targeted health conditions improved their lifestyle behaviours. This complementarity was only statistically significant for smoking, which reduced by 0.7 cigarettes per person per day, equal to 18% of the mean. We investigate whether this change was attributable to the QOF by testing for other discontinuity points, including the introduction of a smoking ban in 2007 and changes to the QOF in 2006. We also examine whether medication and smoking cessation advice are potential mechanisms and find no statistically significant discontinuities for these aspects of health care supply. Our results suggest that a general improvement in healthcare generated by provider incentives can have positive unplanned effects on patients’ behaviours.

## Highlights

• Weight control, smoking and drinking can complement or substitute for health care
• Introduction of the Quality and Outcomes Framework acts as a natural experiment
• Individuals with targeted conditions reduced their smoking
• Results are robust to validity tests of the regression discontinuity design
• Financial incentives for healthcare providers have positive spillovers on behaviours

## 1 Introduction

Major non-communicable diseases are the primary cause of death in developed countries. In Europe, the five major non-communicable diseases (diabetes, cardiovascular diseases, cancer, chronic respiratory diseases and mental disorders) account for 86% of deaths (WHO Europe, 2012). These diseases are generally chronic in nature, requiring long-term management. Best practice management and prevention uses both drug treatments and behaviour change interventions targeting smoking, alcohol consumption, diet and physical activity (NICE Guidelines, 2009, NICE Guidelines, 2014, NICE Guidelines, 2015).

The efficacy of this approach depends, in part, on whether receiving health care influences a patient’s decisions to invest in health behaviours. Economic models of health production propose that both medical treatment and an individual’s health behaviours are inputs in the production function for health capital (Becker, 2007, Grossman, 1972). The response of an individual to an increase in treatment provided by the health service depends on whether health care and health behaviours are substitutes or complements in the health production function.

From the individual’s point of view, health behaviours are chosen at the level where the marginal costs equal the marginal benefits of effort. Because healthcare is an additional input, the optimal choice to the individual depends on what she believes about the joint productivity of the two inputs. On the one hand, the individual might believe that improved health care can raise the marginal productivity of her own health behaviour and decide to complement health care with additional effort in healthier behaviours. On the other hand, health care can lower the cost of unhealthy behaviours by compensating for their negative effects. Individuals may therefore reduce their effort in undertaking healthier lifestyles. The relative size of these two effects determines which one prevails over the other.

Kaestner et al. (2014) used a Becker-type health production model (Grossman, 1972) with multiple types of health investment to examine the effect of a reduction in the price of statins on health behaviours. Their model predicted an ambiguous relationship between statins and health behaviours. The direction of the effect depends on whether the pure income effect, leading to more consumption of both statins and healthier behaviours, prevails over the substitution effect.

Investigation of the causal effect of health care on health behaviours is difficult in observational settings because treatment is not assigned randomly. Disease, and the causes of disease, determine treatment assignment and may also influence health behaviours. Health behaviours influence disease occurrence and therefore treatment assignment, creating a selection bias if we compare treated and untreated samples.

A few studies have used instrumental variable models to overcome the treatment endogeneity problem. Kaestner et al. (2014) used the gradual penetration of statins in the U.S. market since their introduction in 1987 as an instrument for statin use. They used the Framingham Heart Study and found that statin use was associated with a small increase in Body Mass Index (BMI) and larger increases in the probability of being obese. They found that an increase in statins was associated with a 0.3–0.5 point increase in BMI for females and males and an increase of 15% of the mean in moderate alcohol consumption by males. Their results provide evidence for a strong substitutability of healthier behaviours and healthcare. However, they found no consistent evidence of a decrease in smoking as a result of statin use.

Fichera and Sutton (2011) used three cross-sections of the Health Survey for England to determine the effect of lipid-lowering drugs and smoking cessation advice on quitting smoking behaviour. In a trivariate probit regression they adopted an exclusion restriction involving the individual’s level of cholesterol and type of heart disease. They found that prescription of lipid-lowering drugs increased the probability of smoking cessation by 20–28 percentage points in patients with cardiovascular diseases. However, the assumption of no direct effect of the type of heart disease on behaviour was not testable in cross-sectional data.

Schneider and Ulrich (2008) used two waves of the German Socio-Economic Panel Study to investigate the relation between the number of doctor visits and a patient’s BMI and smoking behaviour. Although they found complementarity between the visits to the doctor and health behaviours, their identification strategy relied on a number of instruments (specifically stress, economic worries and a regional dummy for living in East Germany) to affect health behaviours but not healthcare utilisation.

In this paper we exploit an exogenous change in the provision of health care. This was caused by a change in the financial incentives for family doctors to provide treatment. These highly powered incentives, the Quality and Outcomes Framework (QOF), were initiated in April 2004 and aimed to improve the quality of primary care through financial rewards for achievement against a number of indicators of health care provision and health outcomes. We observe the effects of the QOF using data from the Health Survey for England (HSE) (1997–2009). This household survey gathers data on health behaviours and health status from repeated cross-sections of the English population.

We exploit the known date of introduction of the QOF with a regression discontinuity approach. We address treatment endogeneity in two ways: i) the introduction of the QOF induces a step change in treatment that is independent of individual’s behaviour; and ii) as the interview date is independent of the QOF and the sample is randomly drawn from the population, so are the unobserved attitudes towards health of these individuals.

A systematic review by Gillam et al. (2012) reported that the QOF improved care quality with enhanced processes and intermediate outcomes for most of the health conditions that it targeted. In a Becker-type model such as the one by Grossman (1972), the value of a marginal investment in healthier behaviours has increased, because improved health care induces a higher probability of survival to enjoy the benefits. This is similar to the “competing risk of death effect” described by Kaestner et al. (2014). However, if healthcare and health behaviours are substitutes in health production then the model predicts lower investments in healthier behaviours. Kaestner et al. (2014) label this the “technical substitution effect”. Therefore, the effect of the QOF-induced increase in the supply of healthcare on health behaviours is an empirical question.

We examine the effect of the QOF on three health behaviours (BMI, smoking and alcohol consumption) in a population of individuals with health conditions targeted by the QOF. We find evidence of complementarity between healthcare and healthier lifestyle choices with a statistically significant average reduction of 0.7 cigarettes per person per day (equivalent to 18% of the mean). We expose our analysis to a battery of robustness checks investigating the sensitivity of the results to the definition of the discontinuity point. Our main results remain unchanged.

We then attempt to determine some potential mechanisms through which the QOF has improved lifestyle behaviours examining the role of medication and smoking cessation advice, which are both measured in the HSE. We do not find any statistically significant discontinuities in medication and smoking cessation advice. This suggests that it was the wider improvements in health care induced by the QOF identified in previous studies (see for example, Gillam et al., 2012; and Sutton et al., 2010) that may have influenced individual’s health behaviours.

The paper is structured as follows. The QOF is described in section 2. The data and descriptive statistics are outlined in section 3. Section 4 contains a graphical analysis. Section 5 describes the empirical strategy and section 6 discusses the results. Section 7 concludes.

## 2 The Quality and Outcomes Framework (QOF)

On the 1st of April 2004, the UK National Health Service introduced a new pay-for-performance scheme for family doctors. This new program, the QOF, encouraged improved treatment and management of specific conditions and increased doctors’ incomes by 25% (Review Body, 2008).

Each financial year running from 1st of April, practices are rewarded on four quality domains: clinical, organisational, additional services and patient experience (Roland, 2004). Each domain contains several quality indicators. Achievement of these indicators provides practices with points, which are converted to income depending on the size of the practice population and disease prevalence rates. In the first two financial years of the program, the clinical domain contained up to 550 points. In 2004/05 the price per point was £75, offering a maximum of about £41,250 for an average practice. In 2005/06 the price per point was raised to £125 amounting to a maximum income of £68,750.

In this paper we consider seven disease areas and18 performance indicators present in the QOF since its introduction in 2004/05 (see description in Table A.1). These indicators accounted for 25% (139/550 points) of the total reward available for clinical care up to 2005/06. Although from the 1st of April 2006 the total number of QOF points changed as new indicators were introduced, the proportion of total points available for our incentivised indicators remains unchanged.

These 18 indicators incentivise control and management of conditions such as asthma, high blood pressure, coronary heart diseases, diabetes, stroke and mental health. Amongst directly incentivised activities, the QOF includes recording of smoking and BMI (for asthma and diabetes) and the provision of smoking cessation advice. In the period we consider there was no direct incentive for reducing alcohol consumption or BMI. However, NICE guidelines on management of cardiovascular diseases and diabetes include nutritional advice, and other behavioural change interventions relating to reduction of alcohol and cigarette consumption (NICE Guidelines, 2009, NICE Guidelines, 2014, NICE Guidelines, 2015). It is reasonable to suppose that in striving to meet QOF indicators in these conditions providers may more consistently apply best practice management and not only improve directly incentivised treatments.

Most of the literature on the impact of the QOF reports that the program improved quality in the targeted clinical areas, but after 2005/06 it reached a plateau (see for example, Campbell et al., 2009 and Doran et al., 2011). However, almost all of the literature on the effects of the QOF relies on data recorded and/or reported by practices and is therefore susceptible to changes in recording behaviour and/or reporting bias (see Gravelle et al., 2010). There is very little evidence on how the QOF has affected patients, particularly with respect to their health behaviours and their health outcomes (Gillam et al., 2012, Sutton et al., 2010). But this is an important policy issue as the effects of provider incentives may be crowded-out by deteriorations in individuals’ health behaviours.

## 3 Data and descriptive statistics

The Health Survey for England (HSE) comprises annual cross-sectional surveys beginning in 1991 and contains information on diagnoses, health behaviours and prescribed medicines. It is designed to be nationally representative of the English adult population with regard to age, gender, geographic area and socio-demographic circumstances. We use twelve years of data from 1997, after which income information was collected. We did not require ethical approval for this study as we use anonymised, publicly available observational data on individuals.

The HSE contains a “core” sample that is repeated each year and a “boost” sample on subjects of special interest that vary each year. The “core” part includes questions on general health and psychosocial indicators, smoking, alcohol, demographic and socio-economic indicators. The HSE contains information on the medicines that individuals have been prescribed. Respondents are asked whether they are taking any prescribed medication and, if so, the British National Formulary codes of all medications are recorded by the nurse from the packets or bottles. We consider prescription of any drug that is directly incentivised in the QOF for the targeted conditions. As listed in Table A.1, the drugs directly incentivised in the QOF and recorded in the HSE are: lipid-lowering drugs, diuretics, beta-blockers, vasodilators, calcium blockers, anticoagulants and anti-platelet drugs. Smokers in the HSE are asked whether they have been given smoking cessation advice by a medical practitioner and if so, whether such advice was delivered within the past 12 months. As the smoking cessation variable was not recorded in 2000, 2001 and 2002, we use a multiple imputation procedure to account for missing observations (Rubin, 1987). We perform three sensitivity tests on the imputation models by excluding any time trend, by including a linear time trend and, finally, by including a second order polynomial of the time trend. More details on this process are available from the authors on request.

In each year of the “boost” sample, the HSE focuses on specific demographic groups and health conditions. In each year of the survey sampling weights are provided for the over-representation of specific boost samples compared to the overall population (see HSE, 2006). We have rescaled these sampling weights by the mean of the weight in each year.

We select a sample of individuals reporting at least one condition incentivised by the QOF. The health conditions recorded in the HSE related to the seven disease areas targeted by the QOF are: cancer, diabetes, other endocrine problems, mental health, stroke, heart attack/angina, hypertension/high blood pressure, bronchitis, asthma, and other respiratory problems. See Table A.1 for a full description of the disease areas and indicators incentivised by the QOF. The survey contains information on health outcomes and behaviours. We focus on BMI, the average number of cigarettes smoked per day and the frequency of alcohol drinking because they are the only health behaviours consistently measured across survey waves. BMI is defined as the individual’s body mass (in kilograms) divided by the square of her height (in metres). It is the net of energy intake (diet) and energy expenditure (determined by physical activity and other metabolic factors). We believe it is more relevant to use BMI as a proxy for diet and physical activity because it reflects energy balance. This makes it a good proxy of diet and exercise behaviours. For the analysis of BMI, we include only feasible values and trim the bottom and top 5% of the distribution.

In each wave of the survey, respondents are asked whether they smoke and, if so, the average number of cigarettes smoked in a day. Non-smokers are coded as having zero consumption of cigarettes per day.

HSE respondents are also asked about the frequency of their alcohol drinking. Responses range from a scale of one (i.e. once every couple of months or once/twice per year) to five (i.e. almost every day). We dichotomise this variable with a value of one for daily alcohol consumption and zero for any other frequency of consumption. One limitation of our analysis is that we only have measures of the frequency and not the intensity of alcohol consumption (Berggren and Sutton, 1999).

As covariates we include demographic variables, education, region of residence, and income. The demographic variables include gender, age, number of children, and marital status. We also consider an indicator of formal education. Household income is equivalised by household size and deflated using the consumer price index with 2005 as the base year.

Table 1 displays comparisons of health behaviours and individual characteristics two years before and after the QOF was introduced. We choose a relatively small interval around the introduction of the QOF in order to test the assumption that individuals to the left and right of the cut-off date are similar in their baseline covariates. Later on, we will show that the optimal bandwidth is indeed around two years. We have also tested the equality of means at one and three years. These results are not displayed here, but can be made available by the authors upon request.

After the QOF was introduced we find that respondents smoke on average half a cigarette less per day than those interviewed before the QOF.

Most of the individual characteristics are not statistically different before and after the QOF was introduced. However, we find that on average patients interviewed after the QOF was introduced have higher incomes than those interviewed before. We also find that after the QOF there are a higher proportion of patients who are not single. In order to reduce biases from imbalances amongst individuals at large intervals from either side of the cut-off, we control for such characteristics in our empirical strategy.

## 4 Graphical analyses

We start our analysis with a visualisation of the (potential) discontinuity of outcome variables after the introduction of the QOF. In order to do so, we take the average of the outcome variables for each bin:

$$\bar{\text{Y}}=\frac{1}{N_{k}}\sum_{i=1}^{N}{\text{Y}}_{i}\text{I}\left(b_{k}<X_{i}\leq b_{k+1}\right)$$

where Y indicates the health behaviours, *N* indicates the total number of observations and *k* bins of equal width are defined along a range of *X* interview dates. We use the interview date to define a bin as a single financial year. On average each bin contains about 2840 observations. There is a trade-off in the choice of bins size as bins that are smaller will have a higher variance but less bias.

**Fig. A.1:**
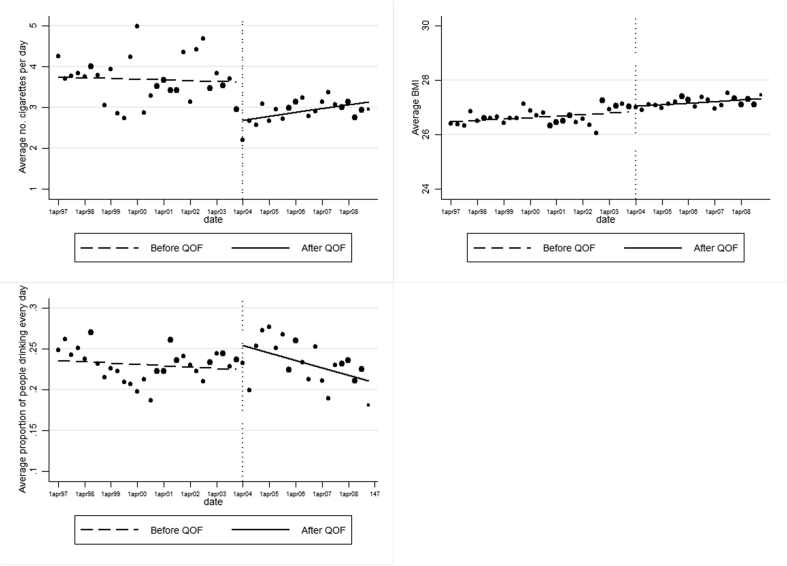
Graphical analysis of changes in outcome variables at the introduction of the QOF (bin width = 3 months). Weighted sample. Each dot indicates the unconditional sample mean for 3 months.

**Fig. A.2:**
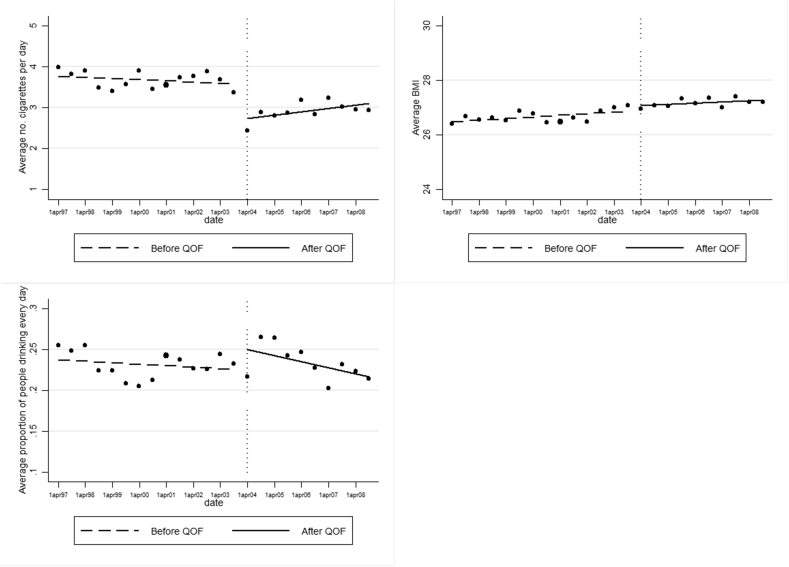
Graphical analysis of changes in outcome variables at the introduction of the QOF (bin width = 6 months). Weighted sample. Each dot indicates the unconditional sample mean for 6 months.

**Fig. 1:**
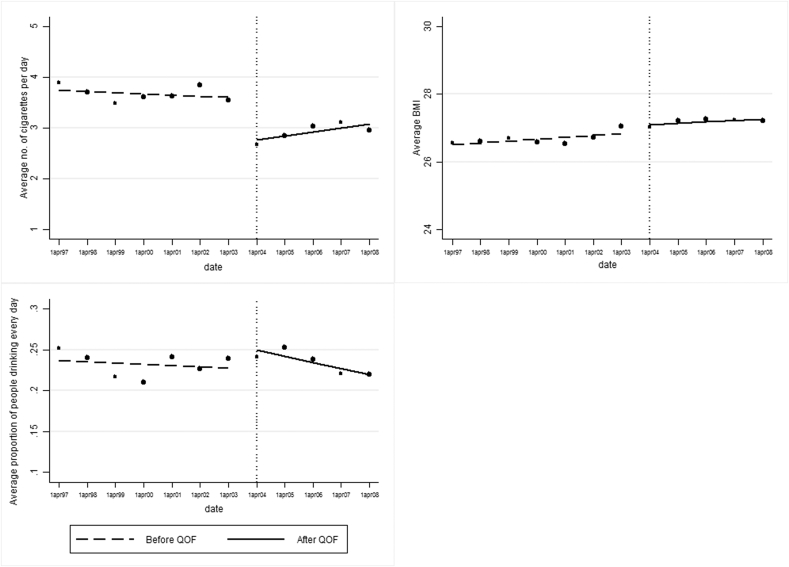
graphical analysis of changes in outcome variables at the introduction of the QOF. Weighted sample. Each dot indicates the unconditional sample mean for a financial year.

Each panel of Fig. 1 plots $\bar{\text{Y}}$ for *k* = *1*, …, *K* against the mid-point of the bins ${\tilde{\text{b}}}_{\text{k}}=\left({\text{b}}_{\text{k}}+{\text{b}}_{\text{k}+1}\right)/2$, as suggested by Imbens and Lemieux (2008). The first panel on the left of Fig. 1 clearly displays a downward jump in the average number of cigarettes smoked per day after the QOF was introduced. There is no clear evidence of any change in BMI or alcohol drinking at this point. In Figures A.1–2 we report the sensitivity of discontinuities to bins that are 3 months and 6 months wide and the results are similar to those in Fig. 1.

Our further analysis seeks to determine whether the reduction in cigarettes smoking observed in Fig. 1 is coincident with the QOF-induced improvement in care.

## 5 Empirical strategy

In order to estimate the effect of the QOF on health behaviours, we use a regression discontinuity design (RDD). This is a variation of the interrupted time series design adopted by Kontopantelis et al. (2014) to examine the effect of the QOF on the quality of primary care for targeted conditions. We argue that better primary care induced by the QOF for people with targeted conditions might induce behavioural changes in their lifestyle choices. Patients with targeted conditions at the margin receive more and better quality treatment after the QOF. These marginal patients might decide to complement or substitute additional treatment with healthier lifestyle choices.

The basic idea behind RDD is that the intervention (i.e. the QOF) is a discontinuous function of a “forcing” variable (i.e. date of interview) being on either side of a fixed cut-off (i.e. the date of introduction of the QOF). To exploit discontinuities in the policy assignment, we assert that individuals on either side of the cut-off are essentially the same in the unobservables or confounders that affect health behaviours. In other words, the unobservable factors (e.g. attitudes towards health) that affect health behaviours are not correlated with the policy itself. This is not an unreasonable assumption in our case as the interview date is not related to the introduction of the policy and the samples are drawn randomly from the national population. Within a sufficiently close interval of the cut-off, conditional on the observed characteristics, the only difference is that people with targeted conditions receive improved treatment after the QOF.

We use a Sharp RD (SRD) design because the intervention applies to all individuals with targeted conditions. We can define:

(1) $$D_{i}=\{\begin{array}{c} 1\;if\;x_{i}\geq c\\ 0\;if\;x_{i}<c \end{array}$$

where *c* indicates 1st April 2004, the cut-off point; and *x*_i_ is the HSE respondent’s date of interview. In the SRD design the assignment to the intervention $D_{i}$ is a deterministic function of the forcing variable $x_{i}$. We apply both a non-parametric and a parametric method to the SRD design, as suggested by Lee and Lemieux (2010). These two methods should be used as complements not substitutes to each other.

### 5.1 Non-parametric method

The first method is based on a local linear regression around the discontinuity. Assume our model is:

(2) $$\;Y_{i}=a+\tau D_{i}+\beta f\left(x_{i}\right)+\varepsilon_{i}\;\mathrm{with}\;D_{i}=1.\left[x_{i}\geq c\right]$$

where *Y*_*i*_ are the health behaviour measures. Since the “forcing” variable *x*_i_ completely determines whether individual *i* is treated, the conditional means are:

$$\mu_{l}\left(x\right)=\underset{x↑c}{\lim}E\left[Y\left(0\right)\text{|}x_{i}=c\right]\;\text{and}\;\mu_{r}\left(x\right)=\underset{x↓c}{\lim}E\left[Y\left(1\right)\text{|}x_{i}=c\right]$$

obtained from two local linear regressions, each at the right, *r*, and left, *l*, side of the cut-off. The SRD design estimator ${\hat{\tau }}_{SRD}={\hat{\mu }}_{r}-{\hat{\mu }}_{l}$ is obtained using standard nonparametric regression methods:

(3) $${\hat{\mu }}_{l}\left(x\right)=\frac{\sum_{i:c-h\leq x_{i}<c}Y_{i}*K_{h}\left(\frac{x_{i}-c}{h}\right)}{\sum_{i:c-h\leq x_{i}<c}K_{h}\left(\frac{x_{i}-c}{h}\right)}\;and\;{\hat{\mu }}_{r}\left(x\right)=\frac{\sum_{i:c<x_{i}\leq c-h}Y_{i}*K_{h}\left(\frac{x_{i}-c}{h}\right)}{\sum_{i:c<x_{i}\leq c-h}K_{h}\left(\frac{x_{i}-c}{h}\right)}$$

where $\left(x_{i}-c\right)$ is the “running” variable defined as the distance of individual *i* to the date of introduction of the QOF. $K_{h}=h^{-1}K\left(.\right)$ is a kernel function distributing weights across the sample points and h=h(n) is a bandwidth parameter determining the width of the kernel. In our empirical specification we use a simple triangular Kernel, e.g. $\text{K}\left(\text{u}\right)=\left(1-\left|\text{u}\right|\right)1_{\left\{\left|\text{u}\right|\leq 1\right\}}$. There are more sophisticated Kernel functions determining weights that decrease smoothly as the distance to the cut-off increases, instead of the 0/1 weights of the triangular kernel. As pointed out by Imbens and Lemieux (2008) results sensitive to more sophisticated kernels are also sensitive to different bandwidths. Therefore, they suggest focusing on the simple triangular kernel and verify the robustness of the results to different choices of bandwidths. This is the approach we take in our analysis. The bigger the sample size, the smaller the weight to observations further from the cut-off. The optimal bandwidth is chosen with the procedure used by Imbens and Kalyanaraman (2009). The estimation has been performed using the *rd* command in Stata (Austin, 2011).

The SRD design allows us to estimate the average treatment effect of the QOF on health behaviours. The crucial identifying assumption for using individuals with targeted conditions interviewed after the QOF as a valid counterfactual for individuals with targeted conditions interviewed before the QOF is that both $E\left[Y\left(0\right)\text{|}x_{i}\right]$ and $E\left[Y\left(1\right)\text{|}x_{i}\right]$ are continuous in $x_{i}$ at *c*. In other words, this means that all unobserved determinants of health behaviours are continuously related to the forcing variable $x_{i}$.

### 5.2 Parametric approach

The second method uses the full sample before and after the QOF was introduced with a polynomial regression in which the equivalent of the bandwidth choice is the choice of the correct polynomial order (see Lee and Lemieux, 2010).

The polynomial regression can be estimated as a pooled OLS regression on both sides of the cutoff point:

(4) $$\;Y_{i}=a+\tau D_{i}+\beta_{1}\sum_{j=1}^{m}f^{m}\left(x_{i}-c\right)+\beta_{1}\left[D_{i}*\sum_{j=1}^{m}f^{m}\left(x_{i}-c\right)\right]+\varepsilon_{i}$$

where *m* = *1,2,3* is the order of the polynomial. We report a number of specifications with different polynomial orders to illustrate the robustness of our results. We use the Akaike Information Criterion for model selection.

### 5.3 Robustness checks

As the continuity assumption of the RDD is not testable, we follow Lee and Lemieux (2010) in using two indirect tests for the validity of this method.

First, we examine whether the observed baseline covariates are “locally” balanced on either side of the cut-off. Intuitively, if RDD is valid, the intervention cannot influence variables not determined by its introduction (for example, the average age and gender composition of respondents). Here we test the assumption of zero effects on those baseline characteristics using the polynomial regression described above with the inclusion of the same control variables used throughout this paper. As a placebo test, we run two separate OLS models just like model (4), but with Y being age and gender as functions of several covariates.

The second validity test looks for jumps at non-discontinuity points. The approach here is similar to the treatment effect literature, as we test for a zero effect in a period when we know the effect should be zero (see Imbens, 2004). We follow Imbens and Lemieux (2008) and perform the first test as follows. We calculate the median of each sub-sample at either side of the cut-off. The median is three years for the sub-sample before the cut-off and 2.4 years for the sub-sample after 1st April 2004. Imbens and Lemieux (2008) suggest the use of the median as a way to increase the power of the test. A “virtual” policy dummy variable indicates for each sub-sample whether observations are above or below the median. This validity test consists of establishing whether the “virtual” policy dummy is statistically significantly different from zero in a set of OLS regressions of the outcome variables. This is why this method is often referred to as the “falsification” or “placebo” test.

As new indicators were introduced in the QOF and thresholds of achievements were changed from 1st April 2006, we use this revision of the program as an additional potential discontinuity point and repeat the analysis of sub-section 5.2. The assumption is that the introduction of new indicators although unrelated to our targeted groups might have generated a shift in individuals’ behaviours. In addition, a smoking ban came into force in England on 1st July 2007 making it illegal to smoke in enclosed public places. Evidence on the effectiveness of smoking bans on active smoking is rather mixed (Callinan et al., 2010). We test whether this reform affects cigarette consumption.

As an additional sensitivity analysis, we investigate whether our results are robust to the inclusion of regions which are only consistently classified from 1998. We also examine whether the reduction in cigarette consumption is driven at the extensive (through quitting) or intensive (through a reduction in consumption amongst smokers) margins.

Finally, the introduction of the QOF has induced improvements in the quality of care for targeted conditions through many aspects of the primary care services (i.e. monitoring of the condition, increased contacts with the doctor, prescribing, lifestyle advice etc.). Some of these services are directly targeted by the policy and some are recorded in the HSE. Table A.1 reports two types of treatment incentivised by the QOF and recorded in the HSE for this population group: prescription of medication and provision of smoking cessation advice. We investigate whether medication and cessation advice jump at the discontinuity point.

## 6 Results

For each health behaviour reported in Table 2, we display five models with different bandwidths: one for the optimal bandwidth; two for multiples of the optimal bandwidth greater than one; and two for multiples of the optimal bandwidth smaller than one. We show that the improvement in primary care quality induced by the QOF is associated with healthier lifestyle behaviours. At the optimal bandwidth we find a decrease in the probability that people with QOF targeted conditions drink every day, a small reduction in BMI (0.8% of the mean) and a reduction in cigarette smoking, but only the latter is statistically significant. The magnitude of the average treatment effect on people with QOF targeted conditions is a reduction of approximately 0.7 cigarettes per person per day (approximately an 18% decline of the mean). We replicate the analysis for multiples of the optimal bandwidth and the results are very similar (with a decline of between 16% and 21% of the mean). The statistical significance at narrower bandwidths is weaker because of the (sub-optimally) smaller sample size.

For each health behaviour reported in Table 3, we display two models: one for the best polynomial order and one for the second-best polynomial order. Using the Akaike Information Criterion values we select the first and second polynomial orders for BMI (AIC = 137060.6 and AIC = 137060.8, respectively); the second and third polynomial orders for alcohol drinking (AIC = 29022.95 and AIC = 29024.35, respectively); and the third and second polynomial orders for cigarettes smoking (AIC = 214921.3 and AIC = 214926, respectively).

The parametric models confirm a statistically significant reduction in cigarette smoking. The magnitude of the average treatment effect ranges between a decline of 0.69 and 0.92 cigarettes per person per day. The coefficient of the parametric regression is slightly different from the non-parametric regression due to missing values for some of the additional covariates (i.e. education, income, marital status and number of children). The inclusion of these covariates is advisable when the parametric regression is estimated over the full sample. As additional sensitivity checks to the parametric regression, we have included just age and gender, and then also all the covariates including regional dummies (consistently available in the HSE from 1998). We have found the coefficient to be very similar to the coefficient in the non-parametric regression. These results are available from the authors upon request.

The results of the first robustness check confirm that the observed baseline covariates are “locally” balanced on either side of the cut-off. There is no statistically significant effect of the QOF on age or gender at any of the displayed polynomial orders. At the optimal polynomial order the effect of the QOF on age is −0.28 (with standard error 0.78) and on female is 0.01 (with standard error 0.02).

The second validity test looks for jumps at other potential discontinuity points. The results presented in Table 4 show no statistically significant effect for placebo reforms.

Other potential discontinuity points could have been changes in the structure of the QOF introduced from 1st April 2006 and the introduction of the smoking ban in England on the 1st of July 2007. In Table 5 we report the effect of the QOF policy change in 2006 on each health behaviour (Model I) and the effect of the smoking ban in 2007 on the number of cigarettes smoked (Model II). Polynomial regressions report no statistically significant effect of the QOF on health behaviours around an interval of the 1st April 2006. Even on the date of the smoking ban in England there is no statistically significant change in smoking behaviour. This confirms results by Jones et al. (2015) using the British Household Panel Survey.

We investigate whether the reduction in smoking is apparent at the extensive or intensive margins. Whilst we find no evidence of a statistically significant reduction in the number of cigarettes smoked by the smokers, we find some evidence of a three percentage point increase in the probability of not smoking. The lack of statistical significance on the intensive margin might be driven by the smaller sample, which only includes smokers.

Finally, we investigate potential mechanisms through which the QOF policy might be associated with changes in healthier lifestyle choices (see Table 6). Medication and smoking cessation advice are two of the mechanisms that are directly incentivised by the QOF and are measured in the HSE. We find no statistically significant effect of the QOF on whether respondents in the HSE sample reported being prescribed medication or receiving smoking cessation advice.

## 7 Conclusions

We find evidence that the introduction of the QOF in April 2004 is associated with a reduction in BMI, frequency of alcohol drinking and cigarette smoking. This effect was only statistically significant for smoking with a reduction of about 0.7 cigarettes per person per day, an 18% decline in mean consumption among the population of individuals reporting conditions targeted by the QOF. This is an expected result given the higher-powered incentives for doctors to treat these individuals and the high health returns to quitting smoking for this population. This complementarity effect of smoking with health care confirms that found by other studies such as Fichera and Sutton (2011) and Schneider and Ulrich (2008). One explanation for this result is that the value of a marginal investment in healthier behaviours is increased because improvements in primary care induced by the QOF generated a higher probability of survival to enjoy the benefits. This is similar to the “competing risk of death effect” described by Kaestner et al. (2014) and is consistent with a Becker-type model such as Grossman (1972) with multiple types of health investment.

The improvements in care induced by the QOF for individuals with the targeted health conditions might include better monitoring of the condition, increased contacts with the doctor, healthcare, and lifestyle advice. A limitation of our analysis is that we cannot determine exactly which intervention incentivised by the QOF induced a change in smoking behaviour. We have explored potential mechanisms by examining the effect of the QOF on prescription and smoking cessation advice and we have not found any statistically significant evidence of discontinuities for these aspects of healthcare supply. One potential limitation is that we had to impute smoking cessation advice, but we do not think this is problematic as the data shows a consistent linear upward trend in the provision of smoking cessation advice both before the year 2000 and after 2002. Our imputation would be invalid if there was a sudden dip in 2000 that then quickly recovered back to the upward trend from 2003 onward. However, more complex causal pathways such as changing social norms around smoking may have influenced results. Social norms may be driving both the policy, e.g. the choice of QOF indicators, and the behavioural response. Although we cannot directly control for changes in social norms, these effects might be captured, at least in part, by the time trends.

The broad range of effects of the QOF, including unintended spillovers on general practice confounds identification of the mechanisms. Doran et al. (2011) find that performance improved in 22 out of 23 incentivised clinical indicators. There is also evidence for better recording of unincentivised activities. Sutton et al. (2010) examined the effect of the QOF on recording of untargeted factors (i.e. BMI and alcohol consumption) of patients with a targeted disease. Using a difference in differences approach with data from over 300 general practices in Scotland, they found substantial spillovers with almost 11-percentage point increase in the recording of untargeted factors induced by the QOF. Nevertheless, despite the limitations regarding the identification of the mechanism, analysis of large-scale natural experiment is important. The financial resources of general practices were increased by 20–30% (National Audit Office, 2008). It is appropriate that we investigate the effect of this major change on all aspects of population health.

The use of financial incentives for health care providers has raised concerns about the effects these will have on patient health behaviours through a patient moral-hazard effect. The results of this paper should alleviate concerns of undesirable crowding-out effects of provider incentives onto individuals’ health behaviours. Rather, they highlight the potential benefits of positive spillovers of such interventions.

## Figures and Tables

**Table A.1:** Description of the Quality and Outcomes Framework disease areas and treatments recorded in the Health Survey for England.

Indicator	Description
ASTHMA 5	The percentage of patients with asthma who smoke, and whose notes contain a record that smoking cessation advice has been offered within last 15 months
ASTHMA 6	The percentage of patients with asthma who have had an asthma review in the last 15 months
BP3	The percentage of patients with hypertension who smoke, whose notes contain a record that smoking cessation advice has been offered at least once
BP4	The percentage of patients with hypertension in which there is a record of the blood pressure in the past 9 months
CHD4	The percentage of patients with coronary heart disease who smoke, whose notes contain a record that smoking cessation advice has been offered within the last 15 months
CHD7	The percentage of patients with coronary heart disease whose notes have a record of total cholesterol in the previous 15 months
CHD9	The percentage of patients with coronary heart disease with a record in the last 15 months that aspirin, an alternative anti-platelet therapy, or an anti-coagulant is being taken (unless a contraindication or side effects are recorded)
CHD10	The percentage of patients with coronary heart disease who are currently treated with a beta blocker (unless a contraindication or side-effects are recorded)
CHD11	The percentage of patients with a history of myocardial infarction (diagnosed after 1 April 2003) who are currently treated with an ACE inhibitor
DM2	The percentage of patients with diabetes whose notes record BMI in the previous 15 months
DM4	The percentage of patients with diabetes who smoke and whose notes contain a record that smoking cessation advice has been offered in the last 15 months
DM5	The percentage of diabetic patients who have a record of HbA1c or equivalent in the previous 15 months
DM15	The percentage of patients with diabetes with proteinuria or micro-albuminuria who are treated with ACE inhibitors (or A2 antagonists)
MH2	The percentage of patients with severe long-term mental health problems with a review recorded in the preceding 15 months. This review includes a check on the accuracy of prescribed medication, a review of physical health and a review of co-ordination arrangements with secondary care
STROKE2	The percentage of new patients with presumptive stroke (presenting after 01/04/03) who have been referred for confirmation of the diagnosis by CT or MRI scan
STROKE4	The percentage of patients with a history of TIA or stroke who smoke and whose notes contain a record that smoking cessation advice has been offered in the last 15 months
STROKE9	The percentage of patients with a stroke shown to be non-haemorrhagic, or a history of TIA, who have a record that aspirin, an alternative anti-platelet therapy, or an anti-coagulant is being taken (unless a contraindication or side-effects are recorded)

**Table 1:** Comparisons of outcomes and covariates before and after the introduction of the QOF

	Before	After	p-value of difference	N
**Outcomes**				
Cigarettes smoked per day	3.80	3.20	0.004***	10,924
Drinking alcohol daily^†^	0.24	0.24	0.916	9804
Body mass index (kg/m2)	26.9	27.0	0.265	8978
**Covariates**				
Female^†^	0.53	0.54	0.863	13,330
Age	55.8	55.9	0.731	13,330
Formal educational qualification^†^	0.64	0.62	0.374	11,236
Children	0.37	0.33	0.403*	11,236
Cohabiting/ever married^†^	0.59	0.61	0.033**	11,236
Ln(equivalised income)	9.72	9.78	<0.001***	11,236

Notes: Sample sizes of the covariates is the maximum attainable when both cigarette consumption and any of the covariates are not missing. Sample size of the outcome variables is conditional on that specific outcome variable not containing missing values. Survey weights applied. *p*-values are obtained from t-tests on the equality of means. ^†^0/1 dummy variable. Samples are within two years before and within two years after the introduction of the QOF. ***p < 0.01, **p < 0.05 *p < 0.10 of difference of means before and after.

**Table 2:** Estimates of the effect of the QOF on health behaviours using local linear regression.

	BMI		Alcohol		No. cigarettes	
	Coeff.	N.	Coeff.	N.	Coeff.	N.
**Model I**:						
With the optimal bandwidth	−0.22 (0.19)	11,270	−0.01 (0.02)	9101	−0.70** (0.28)	19,663
**Model II**:						
With 1.5 times the optimal bandwidth	−0.18 (0.16)	16,441	0.003 (0.02)	14,119	−0.67*** (0.23)	27,269
**Model III**:						
With twice the optimal bandwidth	−0.09 (0.13)	21,397	0.01 (0.02)	17,883	−0.62*** (0.21)	32,102
**Model IV**:						
With 0.3 times the optimal bandwidth	−0.41 (0.35)	3482	0.01 (0.04)	2415	−0.84* (0.49)	5500
**Model V**:						
With 0.4 times the optimal bandwidth	−0.26 (0.31)	4660	0.001 (0.04)	3203	−0.83* (0.43)	7628

Note: optimal bandwidths are as follows: for BMI h_opt_ = 2.5 years; for alcohol h_opt_ = 1.9 years; for cigarettes h_opt_ = 3.4 years. Std. errors in (). Weighted sample. All equations control for age and gender.***p < 0.01; **p < 0.05; *p < 0.1.

**Table 3:** Estimates of the effect of the QOF on health behaviours using polynomial regression.

	BMI		Alcohol		No. cigarettes	
Coeff.	N.	Coeff.	N.	Coeff.	N.
**Model I**:						
With best polynomial order	0.01 (0.11)	25,152	−0.02 (0.02)	27,467	−0.92** (0.37)	31,383
**Model II**:						
With second-best polynomial order	−0.18 (0.17)	25,152	0.004 (0.02)	27,467	−0.69*** (0.19)	31,383

Std. errors in (). Weighted sample. All equations control for age, age squared, gender, educational qualification, number of children, marital status and income.

***p < 0.01; **p < 0.05.

**Table 4:** Test for jumps at placebo discontinuity points.

	Before	After
**Body mass index (kg/m**^2^**)**	−0.69 (0.26)	0.86 (1.11)
*No. observations*	*15,775*	*9239*
**Proportion drinking alcohol daily**	−0.03 (0.03)	0.04 (0.11)
*No. observations*	16,775	10,551
**Cigarettes smoked per person per day**	0.11 (0.48)	1.04 (1.77)
*No. observations*	*19,079*	12,145

Weighted sample. Std errors in (). Table reports the coefficients on dummy variables in an OLS regression indicating two discontinuity points at the median of the sample on either side of the cut-off, namely 3 years before and 2.4 years after the introduction of the QOF. All regressions include: a linear function of month of interview, and its interaction with the placebo reform dummy, and age, age squared, gender, educational qualification, number of children, marital status and income.

**Table 5:** Estimates of the effect of other policies on health behaviours using polynomial regression.

	BMI		Alcohol		No. cigarettes	
Coeff.	N.	Coeff.	N.	Coeff.	N.
**Model I**:						
Changes to the QOF in 2006	0.05 (0.12)	25,152	−0.03 (0.02)	27,467	0.13 (0.27)	31,383
**Model II**:						
Smoking ban in 2007	–		–		0.25 (0.44)	31,383

Note: results refer to best polynomial order. Std. errors in (). Weighted sample. All equations control for age, age squared, gender, educational qualification, number of children, marital status and income.

**Table 6:** Estimates of the effect of the QOF on treatments using polynomial regression.

	Medication		Smoking cessation advice	
Coeff.	N.	Coeff.	N.
**Model I**:				
With best polynomial order	−0.04 (0.04)	23,346	0.02 (0.02)	21,418
**Model II**:				
With second-best polynomial order	0.02 (0.03)	23,346	−0.0004 (0.01)	21,418

Std. errors in (). Weighted sample. All equations control for age, age squared, gender, educational qualification, number of children, marital status and income.
